# Sequential versus standard conditioning in untreated MDS patients with blasts undergoing allogeneic HSCT

**DOI:** 10.1038/s41409-025-02711-1

**Published:** 2025-10-01

**Authors:** Radwan Massoud, Evgeny Klyuchnikov, Normann Steiner, Maroly Bohorquez Manjarres, Gaby Zeck, Rolf Krause, Silke Heidenreich, Claudia Langebrake, Adrin Dadkhah, Ina Rudolph, Rusudan Sabauri, Christian Niederwieser, Tetiana Perekhrestenko, Mirjam Reichard, Mathias Schäfersküpper, Franziska E. Marquard, Sofia Oechsler, Gunnar Weise, Kristin Rathje, Maraike Harfmann, Nico Gagelmann, Catherina Lück, Christine Wolschke, Francis Ayuk, Nicolaus Kröger

**Affiliations:** 1 Department of Stem cell transplantation, University Medical Center Hamburg Eppendorf, Hamburg, Germany; 2 University Hospital of Internal Medicine V (Hematology and Oncology), Medical University of Innsbruck, Innsbruck, Austria

**Keywords:** Myelodysplastic syndrome, Stem-cell research

## Abstract

Myelodysplastic syndromes (MDS) can progress to AML and often require allogeneic hematopoietic stem cell transplantation (allo-SCT). The sequential FLAMSA-FB regimen, featuring a cytoreductive FLAMSA phase followed by fludarabine-busulfan (FB) conditioning, may enhance disease control. We retrospectively analyzed 106 untreated MDS patients with blasts 5–19% at the University Medical Center Hamburg who received either FLAMSA-FB (*n* = 45) or standard conditioning (*n* = 61: Thiotepa-Busulfan (*n* = 30), Fludarabine-Busulfan (*n* = 16), Treosulfan-Fludarabine (*n* = 15)). Median follow-up was 24 months. The FLAMSA group was younger (median age 56 vs. 62, *p* = 0.02), but baseline IPSS risk scores (*p* = 0.16) and donor types (*p* = 0.43) were comparable. Engraftment rates were similar. At two years, overall survival (OS) was 62% with FLAMSA and 68% with standard conditioning (*p* = 0.92), while progression-free survival (PFS) was 56% vs. 59% (*p* = 0.92). Non-relapse mortality (22% vs. 25%, *p* = 0.78) and cumulative incidence of relapse (22% vs. 13%, *p* = 0.12) did not differ significantly, nor did grade II–IV acute graft-versus-host disease (GVHD). Propensity score matching in 18 pairs confirmed no significant differences in OS, PFS, NRM, or CIR. However, moderate-to-severe chronic GVHD was higher with FLAMSA-FB (50% vs. 17%, *p* = 0.04). Thus, FLAMSA-FB did not improve transplant outcomes over standard conditioning but was linked to an increased risk of chronic GVHD.

## Introduction

Myelodysplastic syndromes (MDS) represent a heterogeneous group of hematopoietic disorders that may progress to acute myeloid leukemia (AML) and often warrant allogeneic hematopoietic stem cell transplantation (allo-SCT) to achieve durable remission [1]. MDS predominantly affects the elderly, necessitating regimens with a balanced toxicity and tolerability [2]. While various reduced-intensity and myeloablative conditioning (MAC) regimens have been employed [3–9], the optimal approach in untreated patients remains uncertain. In this context, the sequential FLAMSA-FB regimen, incorporating an initial “induction-like” cytoreductive phase (FLAMSA) followed by a conditioning backbone of fludarabine and busulfan (FB), has been suggested to improve disease control prior to allo-SCT [10].

Although FLAMSA-based regimens have shown promise in high-risk or relapsed/refractory disease, their value in treatment-naïve MDS remains controversial [10–13]. Emerging evidence suggests that upfront transplantation may suffice for lower-burden MDS [14, 15], calling into question whether an “induction-like” phase adds clinical benefit [16–25].

We conducted this study to compare transplant outcomes between FLAMSA-FB and standard conditioning regimens in previously untreated MDS.

## Materials and methods

In this retrospective study, we compared allo-SCT outcomes between FLAMSA-FB and standard conditioning regimens.

### Ethics approval and consent to participate

This study was approved by the Ethics Committee of the University Medical Center Hamburg-Eppendorf (UKE) (reference number: 2022-100940-BO-ff). The study was performed in accordance with the Declaration of Helsinki. Informed consent was obtained from all participants.

All patients who underwent allo-SCT for untreated MDS (with 5–20% bone marrow blasts) at the University Medical Center Hamburg between 2006 and 2024 were included. Myeloablative conditioning regimens were defined according to the working group definition [26]. The FLAMSA-FB regimen included fludarabine (30 mg/m^2^; total dose 120 mg/m^2^), amsacrine (100 mg/m^2^; total dose 400 mg/m^2^), and cytarabine (1 g/m^2^; total dose 4 g/m^2^) from days −11 to −8, followed by a 3-day interval without therapy followed by Busulfan (total dose 6.4 mg/kg) from days −4 to −3, and Fludarabine (30 mg/m^2^; total dose 60 mg/m^2^) on days −4 and −3.

The TB regimen consisted of Thiotepa (5 mg/kg per day; total dose 10 mg/kg) on days −6 and −5, and Busulfan (3.2 mg/kg per day; total dose 6.4 mg/kg) on days −4 and −3.

The Treo-Flu regimen consisted of Treosulfan (12 g/m^2^; total dose 36 g/m^2^) on days −6 to −4, and Fludarabine (30 mg/m^2^; total dose 150 mg/m^2^) on days −6 to −2.

The FB regimen consisted of Busulfan (total dose 6.4 mg/kg) from days −7 to −5, and Fludarabine (30 mg/m^2^; total dose 150 mg/m^2^) on days −7 to −3.

Anti-T lymphocyte globulin (ATLG) (Grafalon®, Neovii, Switzerland) was administered with a test dose of 200 mg on day −4, and the remaining doses were fractionated between days −3 and −1. Thymoglobulin (ATG) doses were fractionated between days −3 and −1. Post-transplant cyclophosphamide (PTCy) was administered as 50 mg/kg/day on days +3 and +4. Post-transplant GVHD prophylaxis involved either Ciclosporine A (CSA) for recipients of matched donors or Tacrolimus (Tac) for recipients of mismatched donors, with Methotrexate 10 mg/m^2^ administered on days +1, +3, and +6. Alternatively, CSA/Tac was combined with Mycophenolate Mofetil from day +1 to day +28 for recipients of matched donors and from day +1 to day +35 for recipients of mismatched donor.

All outcomes were measured from the time of allo-SCT. Our Endpoints were progression-free survival (PFS), overall survival (OS), non-relapse mortality (NRM), and cumulative incidence of relapse (CIR). acute graft versus host disease (aGVHD) and chronic graft versus host disease (cGVHD). aGVHD was graded according to the EBMT definition [27]. cGVHD was graded according to National Institute of Health criteria routinely at every visit after allo-SCT [28]. Neutrophil engraftment was defined as the first three consecutive days with a measure of an absolute neutrophil count >0.5 × 10^9^/L. Platelet engraftment was defined as the first consecutive days with a platelet count >20 × 10^9^/L without transfusion support.

PFS was defined as the duration of survival without relapse or progression, with censoring for patients without these events at last follow-up. OS was defined as death from any cause, while NRM was defined as death without evidence of relapse.

For statistical analysis, Kaplan–Meier methods were employed to estimate probabilities for PFS and OS, with group differences assessed via the log-rank test. Cumulative incidence functions were used to estimate engraftment, CIR, NRM, aGVHD, and cGVHD in a competing risk framework. Specifically, CIR and NRM were treated as competing events, and death without the respective event was considered the competing risk for aGVHD, cGVHD, and engraftment.

For univariate analyses (UVA), continuous variables were categorized. Univariate comparisons were performed using the log-rank test and Gray’s test for cumulative incidences. Multivariate analysis (MVA) was conducted using a Cox proportional hazards model to calculate adjusted hazard ratios and 95% confidence intervals. A *p* value of less than 0.05 was considered statistically significant. MVA using Fine and Gray’s competing risks regression model was performed to identify independent prognostic factors for CIR, GVHD, and engraftment. Variables significant in univariate analysis or clinically relevant were included.

To reduce confounding and improve comparability between treatment groups, we performed propensity score matching (PSM) using the MatchIt package in R. Patients were restricted to those with an Eastern Cooperative Oncology Group performance status of 0 or 1, and haploidentical transplants were excluded to enhance matching quality. A nearest-neighbor matching algorithm with a caliper of 0.25 was applied without replacement. The propensity score was estimated using a logistic regression model, incorporating the following covariates: IPSS score, donor sex, transplant year, donor relationship (related vs. unrelated), and HLA matching status (matched vs. mismatched).

Balance between groups before and after matching was assessed using standardized mean differences, with a threshold of <0.1 considered acceptable. A Love plot and propensity score density plots were generated to visually assess balance improvement.

All statistical analyses were conducted using R (version 3.0, R Development Core Team, Vienna, Austria; https://www.r-project.org/). The following R packages were used: survival, cmprsk, ggplot2, dplyr, survminer, mstate, tableone, forestplot, matchit, and cobalt.

## Results

### Patients donor and transplant characteristics

A total of 106 patients were included in the study, with 45 patients receiving FLAMSA-FB and 61 patients receiving standard conditioning. The median (range) year of transplantation was 2010 (2006–2020) for FLAMSA, 2021 (2018–2024) for TB, 2017 (2000–2022) for FB, and 2019 (2003–2023) for Treo-Flu (*p* < 0.001). The median age at transplantation was 56 years (range, 20–72) in the FLAMSA group compared to 62 years (range, 28–77) in the standard conditioning group (*p* = 0.02).

IPSS risk scores were comparable between groups (*p* = 0.162). In the FLAMSA-FB group, 7% of patients were classified as low-risk, 14% as intermediate-1, 64% as intermediate-2, and 16% as high-risk. In the standard conditioning group, 16% were low-risk, 26% intermediate-1, 41% intermediate-2, and 17% high-risk.

Donor types were distributed similarly between groups (*p* = 0.426). Matched related donors (MRD) were used in 20% of patients in the FLAMSA group and 16% in the standard conditioning group, while haploidentical donors were used in 2% and 10%, respectively. Matched unrelated donors (MUD) accounted for 51% of FLAMSA transplants and 53% in the standard conditioning group, whereas mismatched unrelated donors (MMUD) were used in 27% and 21% of cases, respectively.

Most patients in both groups received ATG/ATLG for in vivo T-cell depletion (84% in the FLAMSA group vs. 89% in the standard conditioning group, *p* = 0.54). PTCY was administered to seven patients (11%) in the standard conditioning group, while none of the patients in the FLAMSA group received PTCY (*p* = 0.02). No patients received a combination of ATG/ATLG and PTCY.

All patient, donor, and transplant characteristics are summarized in Table 1.

**Table 1:** Patients donors and transplant characteristics.

	FLAMSA-FB	Standard	*p*
	*N* (%)	*N* (%)	
Number of patients	**45 (100)**	**61 (100)**	
Patient age median (range)	56 (20–72)	62 (28–77)	**0.024**
Patient sex			0.693
Male	34 (76)	44 (72)	
Female	11 (24)	17 (28)	
Patient CMV serology			0.56
Neg	21 (47)	25 (41)	
Pos	24 (53)	36 (59)	
ECOG			0.259
0	17 (50)	19 (33)	
1	15 (44)	35 (60)	
2	2 (6)	4 (7)	
Donor age median (range)	34 (18–63)	32 (20–66)	0.74
Donor sex			0.136
Male	35 (78)	54 (89)	
Female	10 (22)	7 (12)	
Donor CMV serology			0.297
Neg	19 (42)	32 (53)	
Pos	26 (58)	29 (47)	
IPSS			0.162
Low	3 (7)	9 (16)	
Intermediate 1	6 (14)	14 (26)	
Intermediate 2	28 (64)	22 (41)	
High	7 (16)	9 (17)	
BM blasts at SCT median (range)	12 (5–19)	8 (5–19)	**0.007**
SCT year median(range)	2010 (2006–2020)	2021 (2000–2024)	**<0.001**
Type of allo-SCT			0.426
MRD	9 (20)	10 (16)	
Haplo	1 (2)	6 (10)	
MUD	23 (51)	32 (53)	
MMUD	12 (27)	13 (21)	
MACRIC			0.144
MAC	9 (20)	20 (33)	
RIC	36 (80)	41 (67)	
Conditioning			**<0.001**
FLAMSA BuFlu	45 (100)	0 (0)	
TreoFlu	0 (0)	15 (25)	
BuFlu	0 (0)	16 (26)	
BuTT	0 (0)	30 (49)	
ATG/ATLG			0.54
No ATG/ATLG	7 (16)	7 (12)	
ATG/ATLG	38 (84)	54 (89)	
Type			**<0.001**
ATLG	23 (61)	53 (98)	
Thymoglobulin	15 (40)	1 (2)	
PTCY			**0.019**
No PTCY	45 (100)	54 (89)	
PTCY	0 (0)	7 (11)	
CD34 x 10 x 6 kg median (range)	7 (2–14)	7 (2–19)	0.63

*ECOG* eastern cooperative oncology group performance status, *CMV* cytomegalovirus, *IPSS* international prognostic scoring system, *BM* bone marrow, *SCT* stem cell transplantation, *MRD* matched related donor, *Haplo*, haploidentical donor, *MUD* matched unrelated donor, *MMUD* mismatched unrelated donor, *MACRIC* myeloablative conditioning vs. reduced-intensity conditioning, *BuFlu* busulfan/fludarabine, *BuTT* busulfan/thiotepa, *TreoFlu* treosulfan/fludarabine, *ATG/ATLG* anti-thymocyte globulin/anti-T-lymphocyte globulin, *PTCY* post-transplant cyclophosphamide, *CD*34 CD34+ cell dose infused (×10⁶/kg), *Neg* negative, *Pos* positive, *FLAMSA* fludarabine, cytarabine, amsacrine-based conditioning, *Standard* standard conditioning.

Bold indicates statistical significance, *p* < 0.05.

#### Transplant outcomes

The median follow-up was 24 months (range, 1–240). The univariate analysis is summarized in supplementary Table 1.

#### Engraftment

The median time to leukocyte engraftment was 10 days (7–18) in the FLAMSA group vs. 11 days (8–28) in the standard conditioning group (*p* = 0.6). Similarly, the median time to platelet engraftment was 10 days (5–29) in the FLAMSA group vs. 12 days (7–50) in the standard conditioning group (*p* = 0.3).

#### OS and PFS

**Fig. 1:**
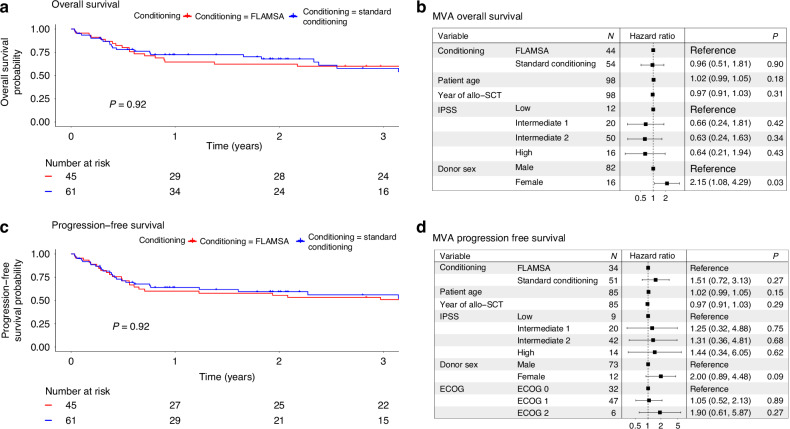
Overall survival and progression-free survival. **a** OS FLAMSA-FB vs Standard. **b** OS MVA. **c** PFS FLAMSA-FB vs Standard. **d** PFS MVA.

OS was comparable between the groups (OS at 2 years: FLAMSA 62% [95% CI 50–78] vs. standard conditioning 68% [95% CI 56–81], *p* = 0.92) (Fig. 1a). Patients transplanted from male donors had higher OS compared to those transplanted from female donors (OS at 2 years: 62% [95% CI 52–74] vs. 47% [95% CI 28–78]). This difference persisted on MVA (HR = 2.15 [95% CI 1.08–4.29] *p* = 0.03) (Fig. 1b).

PFS was comparable on univariate analysis (FLAMSA: 56% [95% CI 43–72] vs Other: 59% [95% CI 48–74], *p* = 0.92) (Fig. 1c). None of the variables affected PFS on UVA.

We observed a trend for inferior PFS in patients transplanted from female compared to those transplanted from male donors (HR = 1.9 [95% CI 0.61–5.87], *p* = 0.27) (Fig. 1d).

### NRM and CIR

**Fig. 2:**
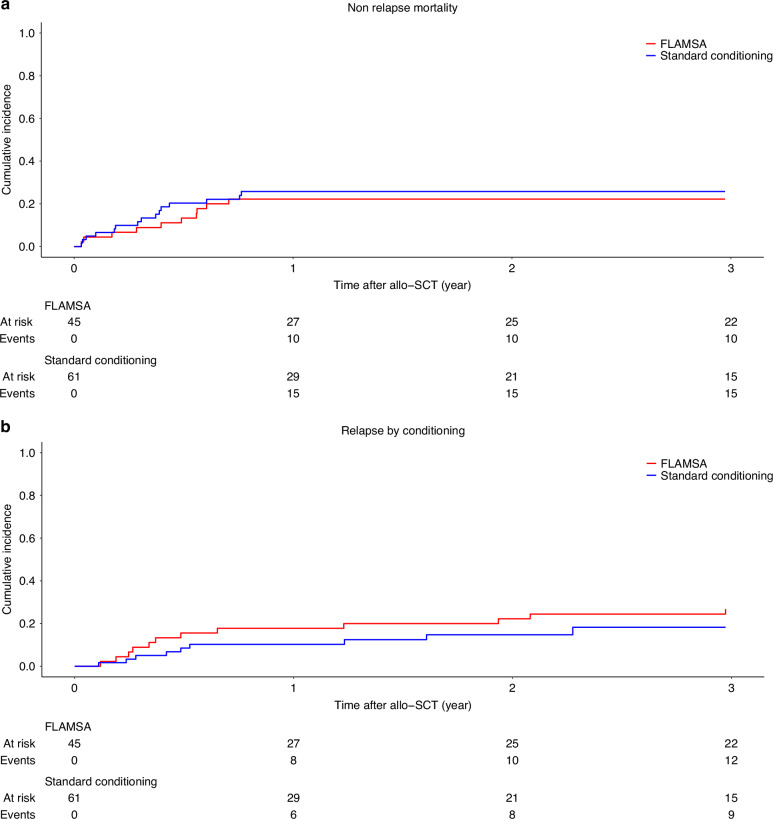
Non-relapse mortality and cumulative incidence of relapse. **a** NRM FLAMSA-FB vs Standard. **b** CIR FLAMSA-FB vs Standard.

NRM was similar (at 2 years: FLAMSA: 22 [11.0–35.0] vs standard conditioning: 25 [15.0–36.0], *p* = 0.78) (Fig. 2a) as was CIR (at 2 years: FLAMSA: 22 [11.0–35.0] vs Other: 13 [6.0–23.0], *p* = 0.12) (Fig. 2b).

### aGVHD and cGVHD

The cumulative incidence of aGVHD grade II–IV and mod/sev cGVHD was also comparable (at 2 years: cGVHD: FLAMSA 40.0 [95%CI 26.0–54.0] vs Other 23.0 [95% CI 13.0–34.0], *p* = 0.06; aGVHD II–IV at day 100: FLAMSA 26% [95% CI 22–50] vs Other 33% [95% CI 21–45], *p* = 0.58).

### Propensity score matching

A total of 18 patients who received FLAMSA and 18 patients who received standard conditioning were successfully matched, while 25 control and 13 treated patients remained unmatched. Prior to matching, significant baseline imbalances were observed between the groups. Following matching, covariate balance improved substantially, with most variables achieving an SMD < 0.1. The improvement in balance across covariates is illustrated in (Supplementary Table 2), Love plot (Supplementary Fig. 1) and the propensity score density plots (Supplementary Fig. 2).

After PSM, OS remained comparable between the groups, with 2-year OS rates of 68% [95% CI 53–86] in the FLAMSA group and 72% [95% CI 59–88] in the standard conditioning group (*p* = 0.62). Similarly, DFS at 2 years was 58% [95% CI 43–78] in the FLAMSA group and 62% [95% CI 49–80] in the standard conditioning group (*p* = 0.79).

At 2 years, the cumulative incidence of NRM was 17% [95% CI 4–37] in the FLAMSA group and 33% [95% CI 13–55] in the standard conditioning group (*p* = 0.43), while the CIR was 17% [95% CI 4–37] and 22% [95% CI 6–44], respectively (*p* = 0.72). The cumulative incidence of grade II–IV aGVHD at day 100 was similar between the groups, at 44% [95% CI 21–66] for FLAMSA and 39% [95% CI 17–61] for standard conditioning (*p* = 0.47). In contrast, the cumulative incidence of moderate/severe cGVHD at 2 years was significantly higher in the FLAMSA group (50% [95% CI 25–71]) compared to the standard conditioning group (17% [95% CI 4–37], *p* = 0.04).

## Discussion

In this retrospective study, we compared transplant outcomes between sequential FLAMSA-FB conditioning and standard conditioning regimens in untreated MDS patients undergoing allo-SCT. Our findings suggest that FLAMSA-FB does not confer a survival advantage over standard conditioning regimens in terms of OS, PFS, NRM, or CIR. Notably, FLAMSA-FB was associated with a significantly higher incidence of moderate-to-severe cGVHD compared to standard conditioning.

The rationale for the sequential FLAMSA regimen is based on its initial “induction-like” cytoreductive phase, which aims to improve disease control before conditioning and transplantation [10]. However, our data indicate that this approach does not translate into improved post-transplant outcomes in untreated MDS. The comparable CIR between FLAMSA-FB and standard conditioning regimens suggests that in treatment-naïve MDS, the additional cytoreduction may not provide a sufficient advantage to justify its use, particularly given its association with increased cGVHD.

Our findings align with the broader literature on pre-transplant cytoreduction in MDS. Over the past two decades, researchers have extensively evaluated whether induction chemotherapy (ICT) before allo-SCT improves outcomes in high-risk MDS. Gerds et al. demonstrated that while bridging with hypomethylating agents showed lower toxicity compared to ICT, adjusted analyses revealed no significant differences in OS or relapse [13]. Supporting this, a large Japanese registry analysis found that upfront allo-SCT without ICT yielded significantly lower CIR, especially in patients with adverse cytogenetics or high IPSS-R scores [14]. Further reinforcing this pattern, an EBMT retrospective analysis showed that while pre-allo-SCT downstaging might offer limited OS benefits in select cases, failure to achieve response was linked to markedly inferior outcomes [15].

The collective evidence suggests that while achieving disease control remains crucial, routine intensive cytoreduction for MDS does not consistently improve post-allo-SCT outcomes compared to less toxic alternatives. This directly supports our observation that FLAMSA did not improve outcomes in untreated MDS, suggesting an added induction-like phase may not benefit this patient population.

Interestingly, FLAMSA has shown efficacy in other contexts. The original report by Schmid et al. included 75 patients with relapsed or refractory AML (R/R AML) and MDS, reporting promising outcomes with a 1-year NRM of 33%, and 2-year OS and LFS of 42% and 40% respectively [10]. Similarly, their study of 103 refractory AML patients undergoing allo-SCT with FLAMSA-RIC and prophylactic DLIs showed meaningful survival rates with a 4-year OS of 32% and LFS of 30% [29]. These findings highlight FLAMSA’s potential utility in R/R AML, particularly with strategies like prophylactic DLI.

The contrast between FLAMSA’s efficacy in R/R settings versus our findings in untreated MDS reinforces the critical role of disease status in conditioning regimen selection. This distinction is further supported by an EBMT ALWP registry study of 1018 patients with R/R AML, which found that FLAMSA-RIC resulted in lower 2-year NRM (7%) and improved OS compared to MAC, though with higher relapse rates [12]. Similarly, our previous study comparing TB, FLAMSA-BuFlu, and Treo-Flu in CMML patients undergoing allo-SCT showed FLAMSA did not improve transplant outcomes [30].

These findings are consistent with a separate EBMT registry study that compared sequential conditioning, MAC, and RIC in 303 patients with MDS and excess blasts. With a median follow-up of 67 months, they found no significant differences in 3-year OS (50%) and RFS (45%) between conditioning regimens [31]. Their MVA identified that outcomes were largely determined by baseline disease risk and patient characteristics rather than conditioning intensity, aligning with our results.

Since FLAMSA is primarily used as a RIC regimen, one might question whether its outcomes are comparable to MAC. However, the prospective randomized EBMT RICMAC study demonstrated similar long-term survival outcomes for RIC and MAC in MDS patients [32].

In light of these findings, FLAMSA-FB does not improve transplant outcomes in untreated MDS, where disease burden is lower than in relapsed or refractory settings. While sequential conditioning has shown benefits in high-risk AML and relapsed MDS, its role in newly diagnosed patients remains uncertain. Our results suggest that immediate transplantation with standard conditioning may be preferable, and further research is needed to refine patient selection criteria for sequential conditioning in this setting.

Our study has several limitations. The retrospective, single-center design introduces selection bias and limits generalizability. While the FLAMSA-FB group had a higher median blast percentage, we adjusted for this by incorporating IPSS into our propensity score and multivariable models. Incomplete cytogenetic and molecular data prevented their inclusion in our analysis, limiting our ability to account for disease biology. Our small sample size and heterogeneity in donor types and conditioning regimens could confound comparisons, though PSM helped reduce these imbalances. Low event counts precluded multivariable analysis for relapse and NRM. We included transplant year in both propensity score and multivariable analyses, while ATG use, though clinically relevant, was already balanced before matching, and its inclusion would have compromised match stability.

FLAMSA-FB does not improve survival or relapse rates in untreated MDS and is associated with higher chronic GVHD risk. Our findings suggest that the added cytoreductive phase offers no advantage in this setting, where immediate transplantation with standard conditioning may be preferable. Prospective studies are needed to confirm these findings.

## Supplementary information

Legends of Supplementary Material

Supplementary Table 1

Supplementary Table 2

Supplementary Figure 1

Supplementary Figure 2

## Data Availability

The analyzed data are available from the corresponding author on reasonable request.
