# Supplementary material for: Sequential versus standard conditioning in untreated MDS patients with blasts undergoing allogeneic HSCT
Source: Bone Marrow Transplant. 2025 Oct 1;60(12):1642–8. doi: 10.1038/s41409-025-02711-1 (PMC12672364; doi:10.1038/s41409-025-02711-1)
Supplement: Supplementary file 1 — Legends of Supplementary Material [file 41409_2025_2711_MOESM1_ESM.docx]

**Supplementary Table 1: Univariate analysis**

Legend: OS: overall survival; PFS: progression-free survival; NRM: non-relapse mortality; CIR: cumulative incidence of relapse; Neg: negative; Pos: positive; FLAMSA: fludarabine, cytarabine, amsacrine-based conditioning; Standard: standard conditioning; ECOG: Eastern Cooperative Oncology Group performance status; CMV: cytomegalovirus; IPSS: International Prognostic Scoring System; BM: bone marrow; SCT: stem cell transplantation; MRD: matched related donor; Haplo: haploidentical donor; MUD: matched unrelated donor; MMUD: mismatched unrelated donor; MACRIC: myeloablative conditioning vs. reduced-intensity conditioning; BuFlu: busulfan/fludarabine; BuTT: busulfan/thiotepa; TreoFlu: treosulfan/fludarabine; ATG/ATLG: anti-thymocyte globulin/anti-T-lymphocyte globulin; PTCY: post-transplant cyclophosphamide; CD34: CD34+ cell dose infused (×10⁶/kg); TB: total body irradiation-based conditioning; FB: fludarabine/busulfan conditioning; Treo-Flu: treosulfan/fludarabine conditioning; MAC: myeloablative conditioning; RIC: reduced-intensity conditioning.

**Supplementary Table 2: SMD before and after matching**

Legend : SMD: Standardized Mean Difference; IPSS: International Prognostic Scoring System; allo-SCT: Allogeneic Stem Cell Transplantation; Related vs Unrelated: Donor relationship status; Match vs Mismatch: HLA matching status. Values reflect balance between treatment groups before and after propensity score matching.

**Supplementary Figure 1**

Legend: Loveplot and Density plots of Propensity Scores
