# Supplementary material for: Sequential versus standard conditioning in untreated MDS patients with blasts undergoing allogeneic HSCT
Source: Bone Marrow Transplant. 2025 Oct 1;60(12):1642–8. doi: 10.1038/s41409-025-02711-1 (PMC12672364; doi:10.1038/s41409-025-02711-1)
Supplement: Supplementary file 2 — Supplementary Table 1 [file 41409_2025_2711_MOESM2_ESM.docx]

|  | **OS** | | **PFS** | | **NRM** | | **CIR** | |
| --- | --- | --- | --- | --- | --- | --- | --- | --- |
|  | **p** | | **p** | | **p** | | **p** | |
|  | **2yr [95%CI]** | **3yr [95%CI]** | **2yr**  **[95%CI]** | **3yr [95%CI]** | **2yr [95%CI]** | **3yr [95%CI]** | **2yr**  **[95%CI]** | **3yr**  **[95%CI]** |
| **Conditioning** | 0.92 | | 0.92 | | 0.79 | | 0.12 | |
| FLAMSA-FB | 62 [50-78] | 60 [47-76] | 56 [43-72] | 51 [38-68] | 22 [11 - 35] | 22 [11 - 35] | 22 [11 - 35] | 27 [15 - 40] |
| Standard | 68 [56-81] | 58 [44-75] | 59 [48-74] | 56 [44-72] | 25 [15 - 36] | 25 [15 - 36] | 13 [6 - 23] | 15 [7 - 25] |
| **Patient age** | 0.22 | | 0.22 | | 0.28 | | 0.63 | |
| ≤64 years | 69 [58-83] | 65 [53-79] | 63 [51-77] | 61 [49-75] | 19 [10 - 30] | 19 [10 - 30] | 17 [9 - 28] | 19 [10 - 30] |
| >64 years | 60 [48-77] | 53 [39-71] | 51 [38-69] | 44 [30-63] | 29 [17 - 42] | 29 [17 - 42] | 17 [8 - 29] | 21 [11 - 33] |
| **Patient CMV serology** | 0.66 | | 0.66 | | 0.87 | | 0.62 | |
| Neg | 73 [61-87] | 65 [52-81] | 59 [46-76] | 56 [43-73] | 20 [10 - 32] | 20 [10 - 32] | 20 [10 - 32] | 22 [11 - 35] |
| Pos | 59 [48-74] | 57 [45-72] | 57 [45-71] | 52 [40-67] | 27 [16 - 38] | 27 [16 - 38] | 15 [7 - 25] | 18 [10 - 29] |
| **Patient sex** | 0.12 | | 0.12 | | 0.18 | | 0.4 | |
| Male | 61 [51-73] | 56 [45-69] | 56 [46-69] | 52 [42-65] | 11 [3 - 25] | 11 [3 - 25] | 25 [11 - 42] | 29 [13 - 46] |
| Female | 77 [62-95] | 72 [56-92] | 62 [45-84] | 57 [40-81] | 28 [19 - 39] | 28 [19 - 39] | 14 [7 - 23] | 17 [9 - 26] |
| **Donor age** | 0.52 | | 0.52 | | 0.49 | | 0.87 | |
| ≤34 years | 66 [55-80] | 59 [47-74] | 55 [44-70] | 53 [41-68] | 22 [12 - 33] | 22 [12 - 33] | 20 [11 - 32] | 22 [12 - 33] |
| >34 years | 64 [51-80] | 61 [48-78] | 61 [48-77] | 54 [41-72] | 26 [14 - 39] | 26 [14 - 39] | 13 [5 - 24] | 17 [8 - 29] |
| **Donor CMV** | 0.16 |  | 0.16 |  | 0.23 |  | 0.97 |  |
| Neg | 74 [63-88] | 66 [53-82] | 64 [51-79] | 61 [48-77] | 16 [7 - 27] | 16 [7 - 27] | 18 [9 - 29] | 20 [10 - 32] |
| Pos | 57 [45-72] | 55 [43-70] | 52 [41-67] | 47 [36-63] | 31 [19 - 43] | 31 [19 - 43] | 16 [8 - 27] | 20 [11 - 32] |
| **Donor sex** | **0.006** | | 0.12 | | 0.05 | | 0.87 | |
| Male | 69 [60-80] | 62 [52-74] | 56 [46-69] | 52 [42-65] | 21 [13 - 30] | 21 [13 - 30] | 17 [10 - 25] | 19 [12 - 28] |
| Female | 47 [28-78] | 47 [28-78] | 62 [45-84] | 57 [40-81] | 35 [14 - 58] | 35 [14 - 58] | 18 [4 - 39] | 24 [7 - 46] |
| **Allo-SCT year** | 0.78 | | 0.78 | | 0.81 | | 0.27 | |
| ≤2017 | 60 [48-74] | 58 [47-73] | 56 [45-71] | 55 [43-69] | 24 [13 - 36] | 24 [13 - 36] | 20 [11 - 32] | 22 [12 - 34] |
| >2017 | 72 [60-87] | 58 [42-79] | 60 [47-76] | 50 [35-71] | 24 [13 - 36] | 24 [13 - 36] | 14 [6 - 25] | 18 [9 - 29] |
| **Type of allo-SCT** | 0.53 | | 0.53 | | 0.27 | | 0.53 | |
| MRD | 72 [55-96] | 72 [55-96] | 67 [48-93] | 59 [40-89] | 21 [6 - 42] | 21 [6 - 42] | 11 [2 - 29] | 16 [4 - 36] |
| Haplo | 43 [18-100] | 43 [18-100] | 43 [18-100] | 43 [18-100] | 57 [13 - 86] | 57 [13 - 86] | 0 [NaN - NaN] | 0 [NaN - NaN] |
| MUD | 69 [57-83] | 58 [45-75] | 55 [43-71] | 50 [37-66] | 22 [12 - 34] | 22 [12 - 34] | 20 [11 - 32] | 24 [13 - 36] |
| MMUD | 59 [42-82] | 59 [42-82] | 59 [43-82] | 59 [43-82] | 20 [7 - 38] | 20 [7 - 38] | 20 [7 - 38] | 20 [7 - 38] |
| **ATG** | 0.98 | | 0.98 | | 0.21 | | 0.12 | |
| No ATG | 57 [36-90] | 57 [36-90] | 57 [36-90] | 46 [24-86] | 43 [17 - 67] | 43 [17 - 67] | 0 [NaN - NaN] | 7 [0 - 30] |
| ATG | 67 [57-77] | 60 [51-72] | 58 [48-69] | 55 [45-66] | 21 [13 - 29] | 21 [13 - 29] | 20 [12 - 28] | 22 [14 - 31] |
| **Conditioning** | 0.69 | | 0.69 | | 0.82 | | 0.6 | |
| FLAMSA-FB | 62 [50-78] | 60 [47-76] | 56 [43-72] | 51 [38-68] | 22 [11 - 35] | 22 [11 - 35] | 22 [11 - 35] | 27 [15 - 40] |
| TB | 81 [68-98] | 61 [39-95] | 67 [51-87] | 56 [36-87] | 17 [6 - 32] | 17 [6 - 32] | 13 [4 - 28] | 17 [6 - 32] |
| FB | 56 [36-87] | 56 [36-87] | 56 [37-87] | 56 [37-87] | 38 [15 - 61] | 38 [15 - 61] | 6 [0 - 26] | 6 [0 - 26] |
| Treo-Flu | 58 [37-91] | 47 [25-87] | 51 [31-86] | 51 [31-86] | 27 [8 - 51] | 27 [8 - 51] | 20 [4 - 44] | 20 [4 - 44] |
| **ECOG** | 0.33 | | 0.33 | | 0.36 | | 0.29 | |
| 0 | 71 [57-88] | 64 [49-83] | 57 [42-76] | 50 [35-70] | 19 [8 - 34] | 19 [8 - 34] | 22 [10 - 37] | 28 [14 - 43] |
| 1 | 68 [56-83] | 61 [47-78] | 63 [50-78] | 59 [46-76] | 24 [13 - 37] | 24 [13 - 37] | 12 [5 - 23] | 14 [6 - 25] |
| 2 | 50 [22-100] | 50 [22-100] | 50 [22-100] | 50 [22-100] | 33 [3 - 70] | 33 [3 - 70] | 17 [0 - 57] | 17 [0 - 57] |
| **IPSS** | 0.93 | | 0.93 | | 0.72 | | 0.75 | |
| Low | 53 [30-95] | 53 [30-95] | 44 [22-88] | 44 [22-88] | 25 [5 - 52] | 25 [5 - 52] | 25 [5 - 53] | 25 [5 - 53] |
| Int1 | 70 [53-93] | 64 [45-90] | 59 [41-86] | 59 [41-86] | 20 [6 - 40] | 20 [6 - 40] | 20 [6 - 40] | 20 [6 - 40] |
| Int2 | 65 [53-80] | 57 [44-74] | 58 [46-74] | 53 [40-70] | 22 [12 - 34] | 22 [12 - 34] | 18 [9 - 30] | 22 [12 - 34] |
| High | 62 [43-91] | 62 [43-91] | 56 [37-87] | 47 [27-82] | 38 [15 - 61] | 38 [15 - 61] | 6 [0 - 26] | 13 [2 - 34] |
| **BM Blasts** | 0.85 | | 0.85 | | 0.63 | | 0.96 | |
| <=10 | 63 [51-78] | 55 [42-72] | 52 [40-68] | 52 [40-68] | 23 [13 - 35] | 23 [13 - 35] | 21 [12 - 33] | 21 [12 - 33] |
| >10 | 67 [55-82] | 65 [52-80] | 63 [51-78] | 55 [43-72] | 24 [13 - 37] | 24 [13 - 37] | 12 [5 - 23] | 18 [9 - 30] |
| **Conditioning Intensity** | 0.53 | | 0.53 | | 0.27 | | 0.95 | |
| MAC | 75 [60-93] | 70 [54-90] | 68 [53-88] | 68 [53-88] | 17 [6 - 33] | 17 [6 - 33] | 14 [4 - 29] | 14 [4 - 29] |
| RIC | 62 [52-74] | 56 [46-70] | 54 [43-67] | 48 [38-62] | 26 [17 - 36] | 26 [17 - 36] | 18 [10 - 28] | 22 [14 - 32] |
| **CD34 x106/Kg** | 0.15 | | 0.15 | | 0.18 | | 0.84 | |
| <=6 | 51 [35-74] | 43 [27-67] | 37 [23-61] | 37 [23-61] | 39 [21 - 57] | 39 [21 - 57] | 21 [8 - 38] | 21 [8 - 38] |
| >6 | 70 [60-82] | 66 [56-79] | 65 [55-77] | 59 [49-72] | 18 [10 - 27] | 18 [10 - 27] | 15 [8 - 24] | 19 [11 - 29] |

Supplementary Table 1: Univariate analysis

Legend: OS: overall survival; PFS: progression-free survival; NRM: non-relapse mortality; CIR: cumulative incidence of relapse; Neg: negative; Pos: positive; FLAMSA: fludarabine, cytarabine, amsacrine-based conditioning; Standard: standard conditioning; ECOG: Eastern Cooperative Oncology Group performance status; CMV: cytomegalovirus; IPSS: International Prognostic Scoring System; BM: bone marrow; SCT: stem cell transplantation; MRD: matched related donor; Haplo: haploidentical donor; MUD: matched unrelated donor; MMUD: mismatched unrelated donor; MACRIC: myeloablative conditioning vs. reduced-intensity conditioning; BuFlu: busulfan/fludarabine; BuTT: busulfan/thiotepa; TreoFlu: treosulfan/fludarabine; ATG/ATLG: anti-thymocyte globulin/anti-T-lymphocyte globulin; PTCY: post-transplant cyclophosphamide; CD34: CD34+ cell dose infused (×10⁶/kg); TB: total body irradiation-based conditioning; FB: fludarabine/busulfan conditioning; Treo-Flu: treosulfan/fludarabine conditioning; MAC: myeloablative conditioning; RIC: reduced-intensity conditioning.
