# Supplementary material for: Sequential versus standard conditioning in untreated MDS patients with blasts undergoing allogeneic HSCT
Source: Bone Marrow Transplant. 2025 Oct 1;60(12):1642–8. doi: 10.1038/s41409-025-02711-1 (PMC12672364; doi:10.1038/s41409-025-02711-1)
Supplement: Supplementary file 3 — Supplementary Table 2 [file 41409_2025_2711_MOESM3_ESM.docx]

| **Variable** | **SMD Before Matching** | **SMD After Matching** |
| --- | --- | --- |
| Distance | 1.3771 | 0.2025 |
| IPSS | 0.514 | 0.0787 |
| Donor Sex | 0.1956 | 0 |
| Year of allo-SCT | 1.3478 | 0.433 |
| Related vs Unrelated | 0.0951 | 0.1329 |
| Match vs Mismatch | 0.2394 | 0.1657 |

Supplementary Table 2: SMD before and after matching

Legend : SMD: Standardized Mean Difference; IPSS: International Prognostic Scoring System; allo-SCT: Allogeneic Stem Cell Transplantation; Related vs Unrelated: Donor relationship status; Match vs Mismatch: HLA matching status. Values reflect balance between treatment groups before and after propensity score matching.
