# Supplementary figures and images for: Sequential versus standard conditioning in untreated MDS patients with blasts undergoing allogeneic HSCT
Source: Bone Marrow Transplant. 2025 Oct 1;60(12):1642–8. doi: 10.1038/s41409-025-02711-1 (PMC12672364; doi:10.1038/s41409-025-02711-1)

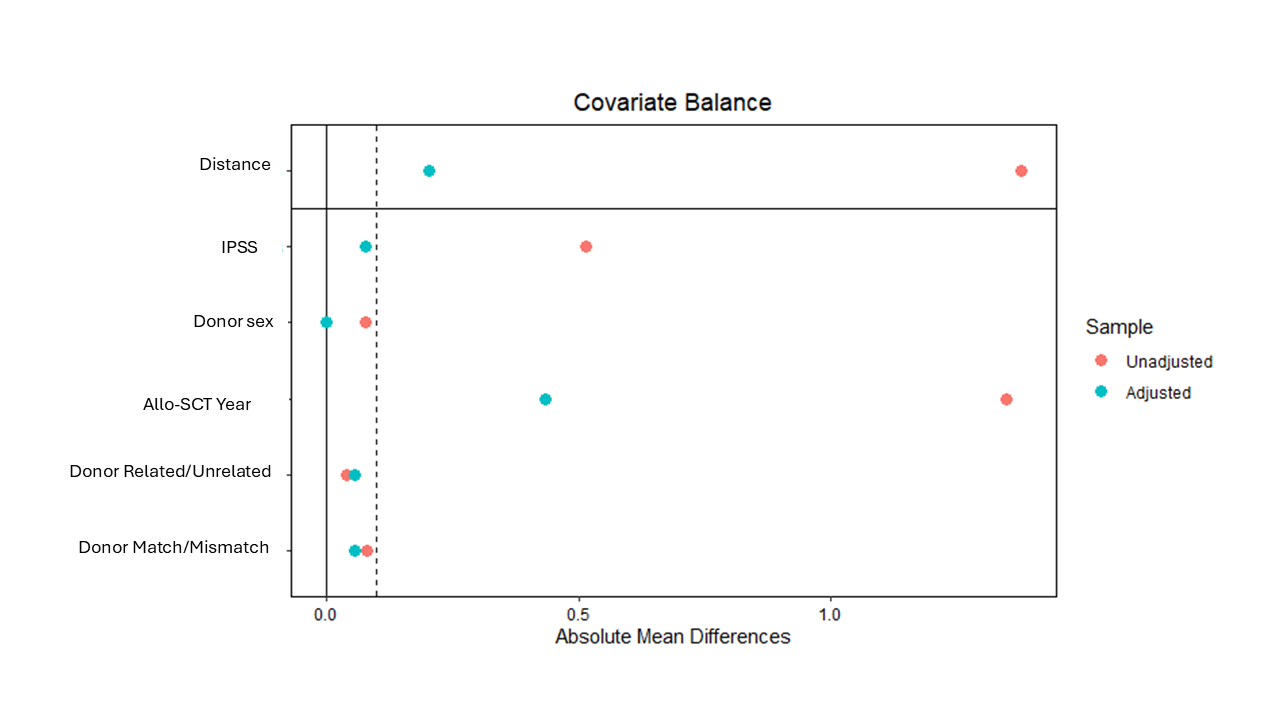

Supplement: Supplementary file 4 — Supplementary Figure 1 [file 41409_2025_2711_MOESM4_ESM.tif]

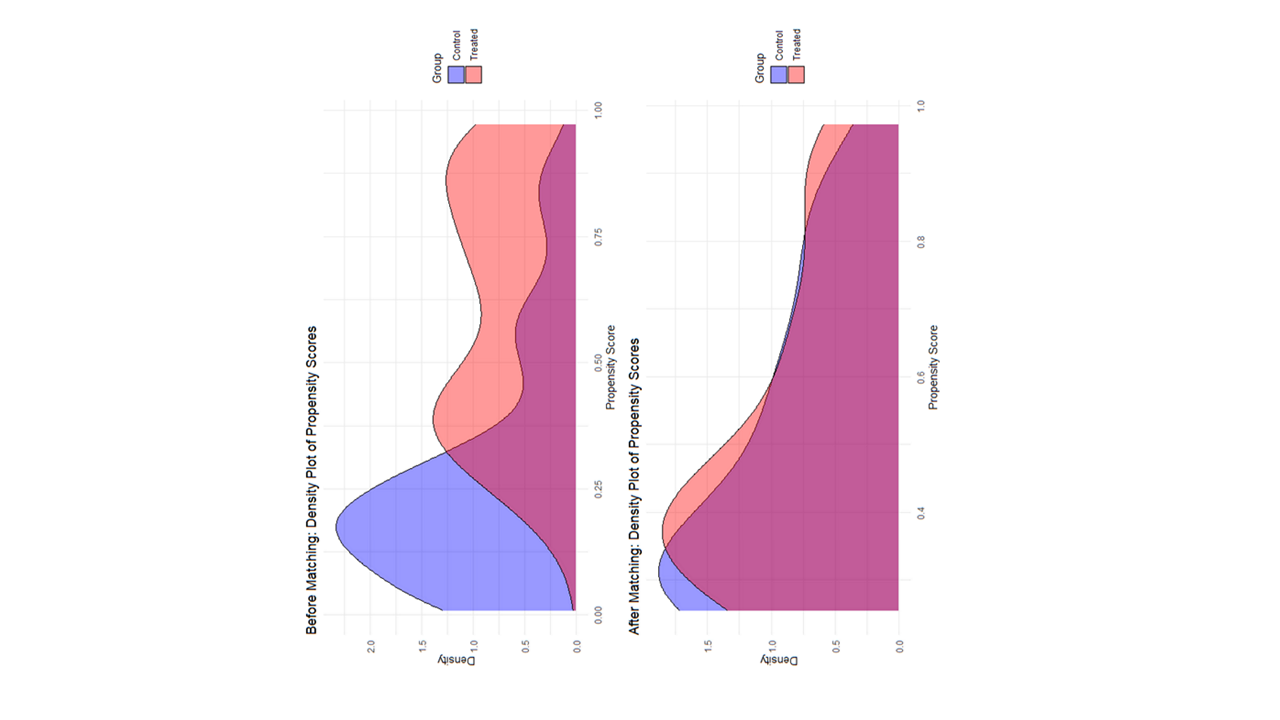

Supplement: Supplementary file 5 — Supplementary Figure 2 [file 41409_2025_2711_MOESM5_ESM.tif]
